# Perceived stress, psychological empowerment and social support among nurses working in psychiatric acute units

**DOI:** 10.1192/j.eurpsy.2023.448

**Published:** 2023-07-19

**Authors:** E. Alexos, G. Koulierakis, A. Zartaloudi

**Affiliations:** University of West Attica, Athens, Greece

## Abstract

**Introduction:**

Perceived stress in the case of health professionals in general and, more specifically, nurses is a frequent phenomenon and occurs when the demands of the clinical environment exceed the available resources in order for nurses to manage the problems that arise.

**Objectives:**

The objective of our study was to investigate the relationship between social support and psychological empowerment with perceived stress of nurses working in psychiatric acute units.

**Methods:**

Our study sample consisted of 153 nurses working in psychiatric acute units, located in Athens area. Participants completed a questionnaire that included a) demographic characteristics, b) the Perceived Stress Scale, c) the Psychological empowerment scale, and d) the Social Support Questionnaire Short Form (SSQ-6).

**Results:**

The majority of our sample were female (62.7%), graduates of Technological Education (47%), married (60.8%), permanent employees (81.7%), working in psychiatric units for over than twenty-one years (34.6%), with an average age of 45.3±6.7 years. The 64.7% of our sample considered the night shift as the most aggravating. The 3 main reasons that caused nurses the greatest stress were (a) lack of staff, (b) dangerousness and (c) workload. Psychological empowerment was positively correlated with social support (r=0.39). When nurses perceived greater social support, were more psychologically empowered as well. Women (b=1.43) compared to men, as well as those who had more years of service in a psychiatric ward (b=0.6), compared to younger employees exhibited a statistically significant higher level of subjective stress. Nurses who considered that night shifts were the most aggravating exhibited more stress (b=1.45). Female nurses (b=3.35), compared to males and those who were scheduled to work more day shifts (b=0.25) exhibited higher levels of psychological empowerment. Married nurses reported higher levels of social support (b=5.66). In contrast, older nurses (b=-0.44), as well as nurses who were scheduled to work more night shifts (b=-1.28), reported statistically significant lower social support.

**Conclusions:**

It is necessary to capture the levels of perceived stress of nurses working in psychiatric acute units and the development of strategies by utilizing the parameters that contribute to reducing stress and act protectively in the workplace, as well as family and wider social environment.

**Disclosure of Interest:**

None Declared

